# Myxoid esophageal liposarcoma: A case report of a rare tumor

**DOI:** 10.1016/j.ijscr.2019.04.001

**Published:** 2019-04-06

**Authors:** Y. Ben Safta, F. Souai, M. Maatouk, A. Zehani, A. Mabrouk, S. Daldoul, S. Sayari, K. Haout, M. Ben Moussa

**Affiliations:** a Surgery A 21 Department, Charles Nicolles hospital, Faculty of Medicine of Tunis, Tunis El Manar University, Tunisia; b Anatomopathology Departement, Rabta Hospital, Faculty of Medicine of Tunis, Tunis El Manar University, Tunisia

**Keywords:** Esophagus, Tumor, Liposarcoma, Lewis Santy

## Abstract

**Introduction:**

Esophageal liposarcoma represent a rare cause of esophageal tumor. According to the literature, since the first case reported in 1983, only 42 cases of esophageal liposarcoma were reported.

**Presentation of case:**

We present a case of liposarcoma in the lower oesophagus treated by surgical resection.

**Discussion:**

Liposarcoma in the oesophagus gastrointestinal tract is an uncommon. An analysis of the literature reports 42 cases.In many cases the diagnosis was established in postoperative period.Surgery is the standard treatment including polypectomy, total or subtotal oesophagectomy.

**Conclusion:**

There is no conventional treatment of this pathology. The curative treatment requires surgical resection or endoscopic approach for selected tumor.

## Highlights

• Esophagealliposarcoma represent a rare cause of esophagealtumor.
• We present a case of liposarcomain the low oesophagus treated by surgical resection.
• There is no conventional treatment of this pathology.
• The curative treatment requires surgical resection orendoscopic approach for selected tumor.

## 1 Introduction

Esophageal liposarcoma represent a rare cause of esophageal tumor, estimated at 0.1–1.5% of esophageal malignant masses [1,2]. It is a malignant sarcoma that most occurs in the retroperitoneum and the deep soft tissues of the lower extremities and the trunk. But It is very rare in the gastrointestinal tract, usually found in and the large bowel or the distal ileum, unusually in the esophagus [3,4]. There were only small cases series have been reported since. Given the scarcity of its occurrence, we report a case of a male who had an esophageal liposarcoma treated with surgical resection managed at our institution and treated with surgical resection.This work has been reported in accordance with the SCARE criteria [5].

## 2 Case report

**Fig. 1:**
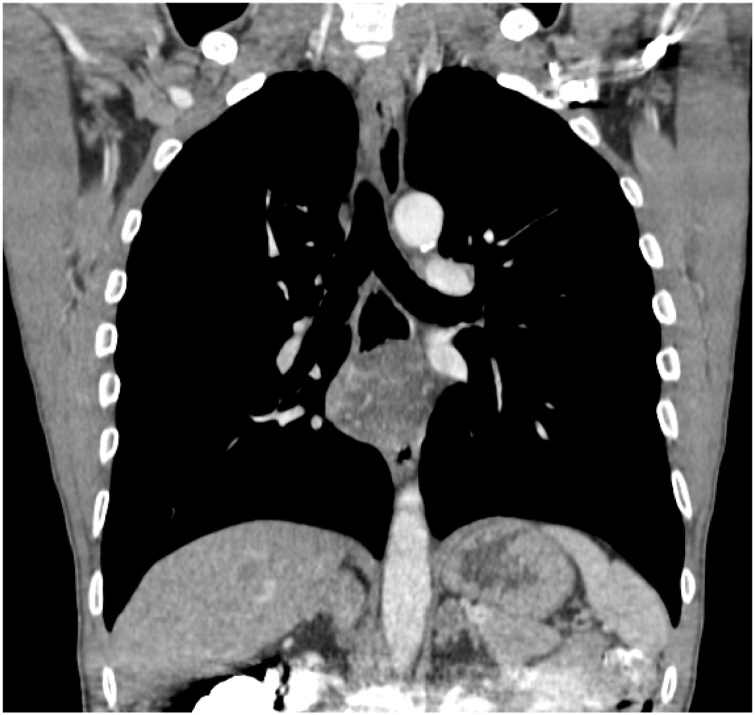
Frontal view of computed tomography of the chest with contrast showing a low oesophagus well-circumscribed mass.

**Fig. 2:**
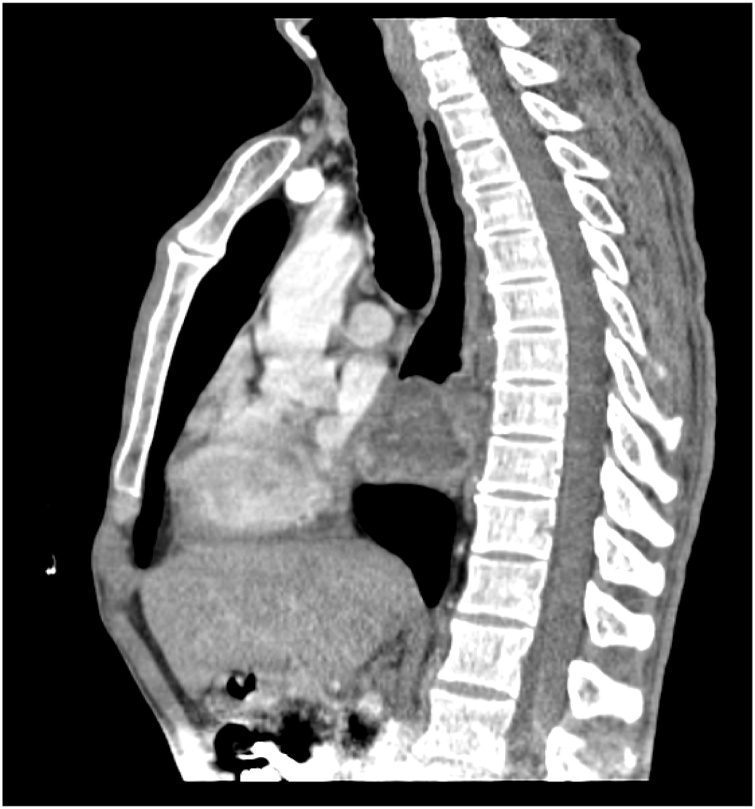
Sagittal view of computed tomography of the chest with contrast showing a low oesophagus well-circumscribed mass.

**Fig. 3:**
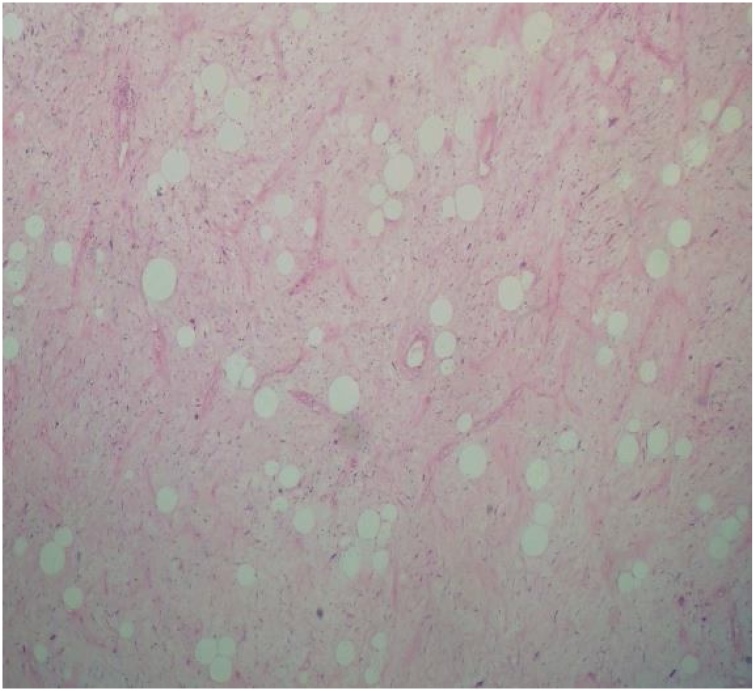
HEX10: malignant adipose tumor proliferation with a mucoid bottom with a network of branched capillaries.

A 44- year- old men presented with progressive dysphagia initially for solid then for liquid food since three years, associated with weight loss of ten kilogram. Physical examination was normal an oesophageal cancer was suspected in first intention, the patient underwent an upper gastro intestinal endoscopy visualising a sub mucosal mass at 26 cm from incisors. The gastro intestinal junction and gastric mucosa were normal. The biopsies of this mass did not show any malignancy sign. A computed tomographic scan of the chest with intra venous contrast and oral opacification showed a well circumscribed mass in the low oesophagus (Fig. 1, Fig. 2). Surgical resection was performed using Lewis Santy approach. On exploration, tumor growth proved to be located in the third lower oesophagus with no invasion of proximal organs and no metastasis. The resection was carcinologic, and a gastric transplant was used. Histologic examination showed myxoid oesophageal amalignant adipose tumor proliferation and mucoid bottom with a network of branched capillaries with negative proximal resection margin and no lymph node infiltration (Fig. 3). The patient had no adjuvant treatment. There was no recurrence after four years of following up.

## 3 Discussion

Liposarcoma in the gastrointestinal tract is an uncommonpathology especially in the oesophagus (1.2% to 1.5% of all gastrointestinal lipomas) [[6], [7], [8]]. Only 42 cases was reported in the literature [9,2].

The first case was described by Mansour et al in 1983 [10]. The most common symptoms are dysphagia, weight loss and regurgitation, more rarely acute gastrointestinal bleeding, achalasia like symptoms [9].

Some patients may presented an uncharacteristic symptoms like chest discomfort, cough, shortness of breath, making the diagnosis more difficult [8]. Due to the mesenchymal nature of liposarcoma it can be confused with the other tumours arising from mucosal or sub mucosal layer like gastrointestinal tumour or lipomas [11]. That’s why para clinical exams must be associated to found the diagnosis, like esophago-gastroduod-enoscopy, computerized tomography scans and magnetic resonance imaging.

The combination between the CT scans and the MRI can help, by evaluating the fat component of the tumour, to differentiate between lipoma with 100% fat content and liposarcoma with less than 75% of fat content [2]. In many cases the diagnosis was established in postoperative period like in our case where the pre-operative investigations did not identify the nature of the mass. Eighty percent of oesophageal liposarcoma were located in the cervical portion of the oesophagus. The majority of lesions cited in the literature were polypoid (78% of cases) [9]. In the present case the tumour was transmural and located in the lower oesophagus. Oesophageal liposarcoma had four histologic subtypes: well differentiated: the most common subtype, dedifferentiated, myxoid cell, pleomorphic [8]. Surgery is the standard treatment of oesophageal liposarcoma including polypectomy, total or subtotal oesophagectomy were performed in 85% of cases in the literature, endoscopic approach including simple polypectomy in 8,6% and endoscopic sub mucosal dissection in 5,7% of cases [12]. In our case endoscopic resection was not possible due to the transmural nature of the tumour, oesophagectomy was mandatory to have a carcinologic resection. Adjuvant treatment is not recommended for oesophageal liposarcoma because of the acceptable results of surgery [8].

## 4 Conclusion

Oesophageal liposarcoma is a rare disease, there is no conventional treatment for this pathology. The curative treatment requires surgical resection or endoscopic approach for selected tumor.

## Conflicts of interest

No potential conflict of interest relevant to this article wasreported.

## Funding

No source of funding.

## Ethical approval

We have reported a single case with no requirement for ethical approval. This manuscript does not describe a clinical study.

## Consent

Written informed consent was obtained from the patient for publication of this case report and accompanying images.

## Author contribution

Ben safta Y: study concept, Data collection, data analysis or interpretation, writing the paper.

Soui F: study concept, writing the paper.

Maatouk M: study concept, writing the paper.

:data collection.

Zehani A: data collection.

Mabrouk A: data collection, data analysis or interpretation.

Daldoul S: data collection, data analysis or interpretation.

Sayari S: data collection, data analysis or interpretation.

Haouet K: advised and designed the report.

Ben Moussa M: advised and designed the report.

## Registration of research studies

N/A.

## Guarantor

Yacine Ben Safta.

## Provenance and peer review

Not commissioned, externally peer-reviewed.
